# Maturation and Assembly of Iron-Sulfur Cluster-Containing Subunits in the Mitochondrial Complex I From Plants

**DOI:** 10.3389/fpls.2022.916948

**Published:** 2022-05-23

**Authors:** Alicia López-López, Olivier Keech, Nicolas Rouhier

**Affiliations:** ^1^INRAE, IAM, Université de Lorraine, Nancy, France; ^2^Department of Plant Physiology, Umeå Plant Science Centre, Umeå University, Umeå, Sweden

**Keywords:** complex I, mitochondria, iron-sulfur cluster, maturation factors, assembly factors, plants

## Abstract

In plants, the mitochondrial complex I is the protein complex encompassing the largest number of iron-sulfur (Fe-S) clusters. The whole, membrane-embedded, holo-complex is assembled stepwise from assembly intermediates. The Q and N modules are combined to form a peripheral arm in the matrix, whereas the so-called membrane arm is formed after merging a carbonic anhydrase (CA) module with so-called Pp (proximal) and the Pd (distal) domains. A ferredoxin bridge connects both arms. The eight Fe-S clusters present in the peripheral arm for electron transfer reactions are synthesized *via* a dedicated protein machinery referred to as the iron-sulfur cluster (ISC) machinery. The *de novo* assembly occurs on ISCU scaffold proteins from iron, sulfur and electron delivery proteins. In a second step, the preformed Fe-S clusters are transferred, eventually converted and inserted in recipient apo-proteins. Diverse molecular actors, including a chaperone-cochaperone system, assembly factors among which proteins with LYR motifs, and Fe-S cluster carrier/transfer proteins, have been identified as contributors to the second step. This mini-review highlights the recent progresses in our understanding of how specificity is achieved during the delivery of preformed Fe-S clusters to complex I subunits.

## Introduction

**FIGURE 1:**
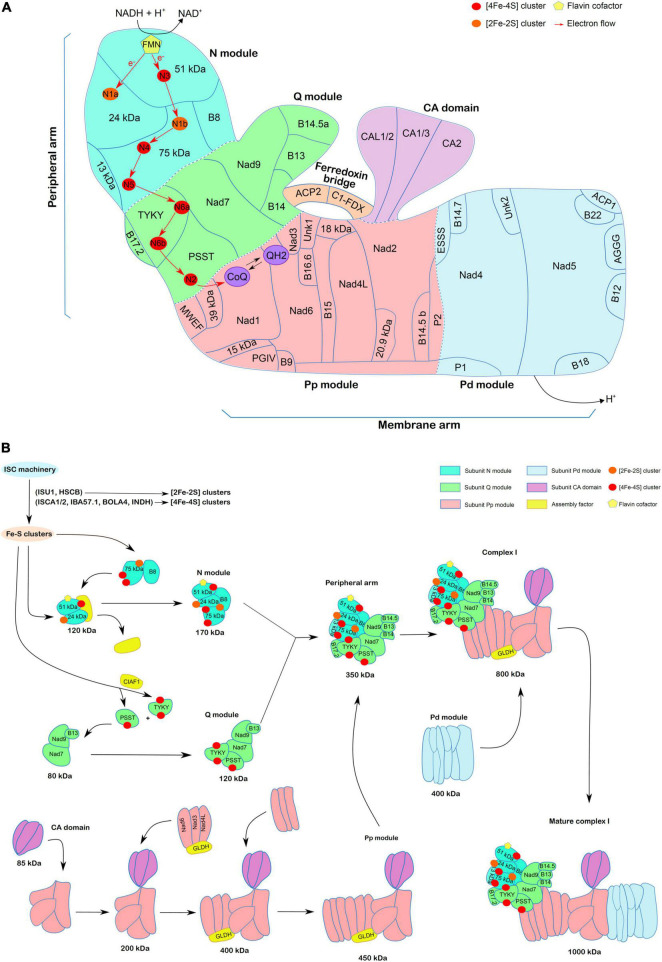
Complex I structure and assembly in plants. **(A)** Schematic representation of complex I structure based on the 3D structure of *Brassica oleracea* complex I (Soufari et al., 2020), including the ferredoxin bridge described in Klusch et al. (2021). Arabidopsis nomenclature is used for the 46 subunits shown here. The CA domain, ferredoxin bridge, modules of the peripheral arm (N and Q modules) and of the membrane arm (Pp and Pd modules) have been represented in different colors. The electron flow from FMN to coenzyme Q *via* the 7 Fe-S clusters present in the different subunits of the peripheral arm is shown. **(B)** Model of the assembly steps of complex I. The figure is adapted from Ligas et al. (2019). Emphasis is made on Fe-S cluster-containing subunits present in the N and Q modules of the peripheral arm. It is thought that they are assembled prior to their insertion in larger subassemblies. Both [2Fe-2S] clusters (orange) and [4Fe-4S] clusters (red) are synthesized *via* the mitochondrial ISC machinery but HSCB and ISCU1/3 scaffold proteins may be sufficient for [2Fe-2S] clusters whereas additional maturation factors such as ISCA1/2, IBA57.1, BOLA4, or INDH and assembly factor (CIAF1) are required at least for some [4Fe-4S] clusters. The N and Q modules, including the Fe-S containing subunits, assemble in parallel following a two-step process forming complexes of 170 and 120 kDa, respectively, and which associate with a few additional subunits in a larger complex of 350 kDa, forming the peripheral arm. In an independent manner, the CA domain (85 kDa) is integrated in a 200 kDa intermediate containing early inserted subunits of the membrane arm. Two further steps are needed to obtain the Pp module of 450 kDa. The L-galactonolactone dehydrogenase (GLDH) assembly factor is present at these steps. The Pp module is then assembled with the 350 kDa to form a 800 kDa complex to which the Pd module is associated to form the mature complex I of 1 MDa. The recently identified ferredoxin bridge is not represented here, but it is likely assembled lately.

Complex I, also defined as NADH-ubiquinone oxidoreductase, is one of the four complexes constituting the mitochondrial electron transport chain. In plants, this enzymatic complex, which catalyzes the NADH-dependent coenzyme Q reduction, is composed of at least 45 subunits including 14 conserved core subunits and additional accessory subunits (Subrahmanian et al., 2016; Meyer et al., 2019; Soufari et al., 2020). As in other organisms, this protein complex adopts a L-shape formed by two main arms (Maldonado et al., 2020). The hydrophobic “membrane arm,” which participates in proton pumping, is embedded in the inner membrane, and is composed of so-called Pp (proximal) and the Pd (distal) domains (Figure 1A). The hydrophilic “peripheral arm” resides in the mitochondrial matrix but is attached to the membrane arm. It is composed of a N module where NADH oxidation takes place, and a Q module where coenzyme Q reduction occurs. Plants, but not yeast or human, have another spherical matrix-exposed domain attached to the membrane arm, known as the carbonic anhydrase (CA) domain (Fromm et al., 2016; Soufari et al., 2020). In addition, a protein bridge connects the Q domain of the peripheral arm (B14 subunit) to the membrane arm (ND2 subunit) and CA domain (γCAL2 subunit) (Klusch et al., 2021). This protein bridge is formed by the acyl-carrier protein 2 (mtACP2/SDAP2) and a ferredoxin-like subunit or complex 1 ferredoxin (C1-FDX). Klusch et al. (2021) proposed that this bridge would regulate complex I activity by setting the angle between its two arms, which would be essential to control the different conformational states of complex I (close and open conformations).

## Modular Assembly of Complex I

The assembly of complex I is a stepwise, precisely orchestrated process. Assembly intermediates (CA, Pd, Pp, Q, and N modules) are initially formed, and then assembled to form the whole holo-complex (Ligas et al., 2019). While the principle of stepwise assembly process is conserved among species, the intermediates are not assembled in the same order in plants as in mammals (Meyer et al., 2019). This process requires several assembly factors, i.e., proteins that contribute to the assembly of the different subunits and domains, but are not part of the final active complex. Although a dozen proteins were identified in mammals, only seven of these exist in plants, namely NDUFAF1 to NDUFAF7 (Meyer et al., 2019). Furthermore, whether they have a similar role remains unclear, notably because they were not found associated with complex I subassemblies so far. Noteworthy, while maturation factors, which are required for the assembly and insertion of iron-sulfur (Fe-S) clusters into functional respiratory subunits, are not part of the final complex I, they are usually not included among assembly factors. Hence, to date, there are only two assembly factors characterized in plants, and in *Arabidopsis thaliana* (Arabidopsis) in particular L-galactonolactone dehydrogenase (GLDH) (Schertl et al., 2012; Ligas et al., 2019) and complex I assembly factor 1 (CIAF1) (Wang et al., 2012; Ivanova et al., 2019).

The peripheral arm is assembled from N and Q modules formed independently (Figure 1B). The N module is formed at least by four subunits. The 51-kDa and 24-kDa subunits in combination with an unknown assembly factor form an assembly intermediate of 120 kDa, to which B8 and 75-kDa subunits associate. It releases the assembly factor to form an assembly intermediate of 170 kDa. The Q module is formed at least by five different subunits: Nad7, Nad9, and B13 that form an assembly intermediate of 80 kDa to which PSST and TYKY subunits are subsequently incorporated thus forming a 120 kDa intermediate (Zhu et al., 2016). Finally, the N and Q modules merge in a 350 kDa assembly intermediate that corresponds to the peripheral arm (Ligas et al., 2019). Some additional subunits (13-kDa, B14, B14.5a, B17.2) are likely added at one of these steps based on the recent *Brassica oleracea* complex I structure (Soufari et al., 2020). All cofactors ensuring electron transfer reactions, i.e., FMN and eight Fe-S clusters, are bound by five subunits of the peripheral arm.

The assembly of the membrane arm also proceeds stepwise. The CA domain (85 kDa intermediate), containing three of the five CAs present in Arabidopsis, i.e., two CAs and one CA-like (CAL) (Fromm et al., 2016), associates with B14.5b, P2, Nad2, and 20.9-kDa subunits leading to the formation of a 200 kDa intermediate. The following assembly of Nad4L, Nad3, Nad6 subunits and of the GLDH assembly factor results in the formation of a 400 kDa intermediate that forms the Pp module of 450 kDa after addition of B9, B16.6, and Nad1 subunits. Association of the Pp module with the peripheral arm results in the formation of an assembly intermediate of 800 kDa (C1*) (Ligas et al., 2019). Finally, the Pd module, which contains among other P1, AGGG, ESSS, B12, B18, Nad4, and Nad5 subunits, is the last functional submodule assembled with the intermediate of 800 kDa to form the whole complex I of 1,000 kDa. GLDH has been identified within the 400, 450, and 850 kDa subcomplexes, but never in the final complex. In its absence, the whole mature complex I is not detectable, whereas the 200 kDa intermediate accumulates (Schertl et al., 2012; Schimmeyer et al., 2016). It was proposed that the final linkage between the Pp and Pd modules is regulated/prevented by GLDH (Soufari et al., 2020). The function of GLDH as an assembly factor of the membrane arm seems independent of its catalytic role in ascorbate synthesis (Schimmeyer et al., 2016). The following sections detail the current state of knowledge about Fe-S cluster synthesis in mitochondria and their insertion into complex I subunits.

## Iron-Sulfur Cluster Assembly and Transfer in Plant Mitochondria

The maturation of mitochondrial Fe-S proteins relies on the iron-sulfur cluster (ISC) machinery and is divided in two basic steps, i.e., Fe-S cluster assembly and transfer (Przybyla-Toscano et al., 2021b).

### *De novo* Assembly of Iron-Sulfur Clusters

The NFS1 cysteine desulfurase is the sulfur donor protein catalyzing cysteine desulfurization. It forms a core complex with ISD11 and acyl-carrier protein (ACP) with the following stoichiometry [NFS1]_2_:[ISD11]_2_:[ACP]_2_ (Boniecki et al., 2017). ISD11 stabilizes NFS1 and contains a conserved Leu-Tyr-Arg motif, which is present in members of the small, eukaryote-specific LYRM protein family (Adam et al., 2006; Wiedemann et al., 2006). ACP interacts with ISD11 through a covalently bound 4′-phosphopantetheine-conjugated acyl chain, further stabilizing the assembly complex. In addition to being essential for Fe-S cluster assembly, ACP provides fatty acid precursors necessary for lipoic acid biosynthesis. In Arabidopsis, there are three mitochondrial ACPs known as mACP1, mACP2, and mACP3 but their respective contribution and interchangeable nature remain unclear (Meyer et al., 2007; Fu et al., 2020).

In the NFS1 reaction mechanism, a conserved Cys becomes persulfidated, and the sulfur atoms are transferred to the ISU/ISCU scaffold protein to be combined with iron atoms to form the Fe-S clusters. Three ISU proteins are present in Arabidopsis and possess the three conserved cysteinyl residues required for Fe-S cluster binding (Léon et al., 2005). While their respective biochemical and structural properties have not yet been compared, expression data indicate that ISU1 is predominant, being constitutively expressed unlike AtISU2 and AtISU3, whose expression seems restricted to pollen (Przybyla-Toscano et al., 2021b). Using mammalian proteins, it was shown that iron binding to ISCU2 drives persulfide uptake from NFS1 before persulfide reduction by FDX2 occurs (Webert et al., 2014; Gervason et al., 2019). Two mitochondrial ferredoxins (MFDX1/2) exist in Arabidopsis, and their redox-active [2Fe-2S] cluster is reduced by a NADPH-dependent mitochondrial ferredoxin reductase (mFDR). Frataxin (FH) is the final key player in this assembly process. It stabilizes NFS1 loops present at the NFS1-ISCU2 interface (Fox et al., 2019), thus accelerating persulfide transfer from NFS1 to ISCU2 (Gervason et al., 2019).

### Transfer of Preformed Iron-Sulfur Clusters to Acceptor Proteins

A chaperone-cochaperone system is required to transfer the preformed [2Fe-2S] cluster bound to ISCU/ISU scaffold proteins either directly to apoproteins (notably respiratory complex subunits) or to other maturation factors. ISCU interacts both with the heat shock protein 70 (HSP70)-type chaperones known as HSCA1/2 in plants (HSPA9 in human) and with the HSP40-type cochaperone HSCB in plants (HSC20 in human) that stimulates the ATPase activity of HSCAs. ATP reloading is performed by MGE1a/b nucleotide exchange factors. HSCB is a key factor recruiting either acceptor proteins or adaptor proteins with a LYR motif (see below) (Maio et al., 2014, 2017).

Based on the yeast model for the ISC machinery, the first receiving maturation factor is glutaredoxinS15 (GRXS15) (Przybyla-Toscano et al., 2021b). Recruitment of GRXS15 by HSCB may rely on a positively charged motif present at the C-terminus of mitochondrial GRXs, but the identified KKX_10_KK in human GRX5 is not conserved as such in plant isoforms, which have more dispersed K residues (Maio et al., 2014). GRXS15 binds a [2Fe-2S] cluster into a homodimer, and transfers it to acceptor proteins such as mFDX1 (Moseler et al., 2015; Azam et al., 2020b). As shown for non-plant proteins (Uzarska et al., 2016), GRXS15 should also bind a [2Fe-2S] cluster in heterodimer with BOLA proteins. A GRXS15-BOLA4 interaction was reported both by yeast-two-hybrid experiments and *in planta* by bimolecular fluorescence complementation assays (Couturier et al., 2014).

Finally, GRXS15 also promotes the formation of [4Fe-4S] clusters onto A-type carrier (ATC) maturation factors referred to as ISCA1a/b and ISCA2 in plants (Azam et al., 2020b). This step consists in the reductive coupling of two [2Fe-2S] clusters into one [4Fe-4S] cluster (Brancaccio et al., 2014; Azam et al., 2020b). In human, the electrons needed for this reaction are brought by FDX2 while another protein named IBA57 assists electron entry into the system (Weiler et al., 2020). IBA57 may also have a role in the Fe-S cluster transfer reaction since a heterodimeric complex formed by human IBA57 and ISCA2 bridges a [2Fe-2S] cluster (Gourdoupis et al., 2018). Further exchange of the [4Fe-4S] cluster bound by ISCA1a/2 heterodimer with other maturation factors named NFU4/5 likely provides specificity toward the numerous recipient mitochondrial Fe-S proteins (Azam et al., 2020a). The observed lethality of *grxs15*, *iba57.1*, and *nfu4 nfu5* Arabidopsis mutant lines demonstrates the critical nature of this pathway for mitochondrial metabolism (Waller et al., 2012; Moseler et al., 2015; Przybyla-Toscano et al., 2022). A complex I-specific maturation factor, INDH, comes into play (discussed below) but its relationship with other ISC maturation factors is unknown.

## Maturation of Iron-Sulfur Cluster-Containing Subunits Present in the N and Q Modules

In complex I, eight Fe-S clusters are bound by five subunits localized in the peripheral arm (Figure 1). In the N module, the 24-kDa subunit binds one [2Fe-2S] cluster, the 51-kDa subunit one [4Fe-4S] cluster and the 75-kDa subunit one [2Fe-2S] cluster and two [4Fe-4S] clusters. In the Q module, PSST binds one [4Fe-4S] cluster and TYKY two [4Fe-4S] clusters (Maldonado et al., 2020). The exact players, mechanisms and specificities by which the different Fe-S clusters are inserted into apoproteins by the ISC machinery only emerged recently.

### The Presence of LYR Motifs in Assembly Factors or Acceptor Proteins Seems to Guide the Insertion of Iron-Sulfur Clusters in Complex I Subunits

In a study performed in human, it was demonstrated that the HSCB cochaperone co-immunoprecipitates all complex I subunits (Maio et al., 2017). Moreover, it was shown that HSCB makes interactions with proteins containing a LYR motif, either present in Fe-S cluster-containing subunits themselves such as SDHB from complex II or in LYRM proteins (Maio et al., 2014, 2017). By recruiting these proteins, HSCB allows substrate discrimination by assisting the guide delivery of nascent [2Fe-2S] clusters bound to the ISCU scaffold to specific subsets of Fe-S recipients. Accordingly, the activity of complex I, and of complexes II and III, is systematically affected in human patients with mutations in the ISCU2, frataxin, ISD11/LYRM4, and FDX2 genes (Selvanathan and Parayil Sankaran, 2022). In studies focusing on complexes II and III, it was shown that Fe-S cluster insertion into target proteins (SDHB or Rieske protein of complex III) is mandatory for the formation of larger complexes (Maio et al., 2014, 2017). This suggests that cluster-free subunits should not assemble into the subcomplexes, and that Fe-S cluster insertion into apo-subunits should not occur in proteins engaged in multipartite protein complex.

**FIGURE 2:**
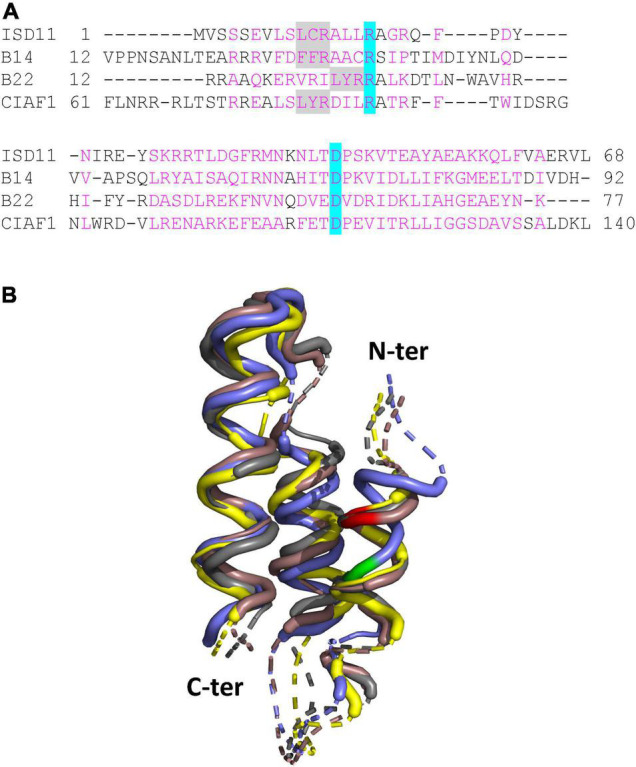
LYRM proteins associated with complex I in plants. **(A)** Structure-based sequence alignment. The alignment was generated with mTM-align (Dong et al., 2018) and manually adjusted to focus on the LYRM common core regions (amino acids are numbered on both sides). We used the structures of the B14 and B22 subunits present in the *Brassica oleracea* complex I cryo-EM structure (Soufari et al., 2020) and structural models of Arabidopsis ISD11 and CIAF1 generated by alphafold (Jumper et al., 2021). Amino acids in magenta correspond to common core regions. The only two conserved residues are highlighted in blue; the LYR motif in gray. Noteworthy, in the B14 protein the FFR motif is degenerated, and the conserved Arginine (R) is not even present in some algal sequences. **(B)** 3D structure superimposition of the core helices. The superimposed structure file in common core regions generated by mTM-align was visualized with Pymol. The ISD11, B14, CIAF1, and B22 proteins are represented in brown, yellow, gray and violet, respectively. Dashed lines represent variable spacing regions. The LYR motif in ISD11, B14, CIAF1 is colored in red, whereas for the B22 protein it is in green and shifted by one helix turn.

In Arabidopsis, the LYRM protein family consists of 10 members (Ivanova et al., 2019). This includes ISD11 and two complex I subunits (B14 and B22). Although their primary sequences are very divergent, they adopt a similar core 3D structure formed by three α-helices (Figure 2) and all interact with ACPs (Angerer, 2013). The rest supposedly represents assembly factors for mitochondrial complexes (Maio et al., 2014, 2017; Ivanova et al., 2019). A recent study highlighted a possible role of the LYRM, plant-specific CIAF1 assembly factor for Fe-S cluster maturation of TYKY. A specific interaction of CIAF1 with TYKY was observed using yeast-two-hybrid assays (Ivanova et al., 2019). Moreover, a 650 kDa intermediate representing the fully assembled membrane arm accumulates in Arabidopsis *ciaf1* knock-down mutants as in mutants for the 24-kDa, 51-kDa, 75-kDa, and PSST subunits (Ivanova et al., 2019; Ligas et al., 2019). All these mutants do not assemble the whole complex I or the supercomplex I + III (Ivanova et al., 2019; Ligas et al., 2019). Arabidopsis *ciaf1* mutants accumulate also 800 kDa intermediates. Therefore, it is possible that, in this case, cluster-free subunits assemble into larger subcomplexes. Alternatively, the remaining CIAF1 protein allows to some extent Fe-S cluster insertion in TYKY, or TYKY maturation also occurs without CIAF1. Interestingly, plant TYKYs possess a strictly conserved LYR motif at the C-terminus that might be sufficient for Fe-S cluster insertion. The 75-kDa subunits from plants also contain a conserved, internal VYR motif unlike the three remaining Fe-S cluster binding subunits.

### Additional Candidate Maturation Factors for the Formation and Insertion of [4Fe-4S] Clusters

Since most Fe-S clusters in complex I are of the [4Fe-4S]-type, the question arises whether the reductive coupling of two [2Fe-2S] clusters donated by ISCU operates despite the existence of several specific maturation factors (ISCA, IBA57, BOLA, NFU4/5, and INDH protein families), which are relevant for direct [4Fe-4S] cluster insertion. This possibility is reinforced by the observation that ISCA1, IBA57, and NFU1 co-immunoprecipitates with HSCB in human (Maio et al., 2017). Moreover, while the [2Fe-2S] containing NDUFV2 (24-kDa ortholog) interacted with HSC20/HSCB, HSPA9/HSCA, and ISCU in a single complex, NDUFS8 (TYKY ortholog) and NDUFS1 were found in higher molecular weight complexes (Maio et al., 2017), possibly including some [4Fe-4S] transfer proteins.

Additional evidence for the contribution of these late-acting maturation factors come from the biochemical phenotypes of human patients or Arabidopsis mutated lines. In human, mutations in *NFU1*, *BOLA3*, *IBA57*, *ISCA2*, and *ISCA1* genes lead to Multiple Mitochondrial Dysfunctions Syndromes 1 to 5 (MMDS1 to MMDS5) respectively (Selvanathan and Parayil Sankaran, 2022). For *BOLA*3, *IBA*57, and *ISCA*1, gene depletion in human cell lines or mutations in human patients consistently lead to defects in complex I assembly/activity, in complex II activity and in enzymes that depend on lipoic acid, the synthesis of which requires the [4Fe-4S]-containing lipoate synthase (Sheftel et al., 2012; Haack et al., 2013; Debray et al., 2015; Torraco et al., 2018). Although the corresponding Arabidopsis mutants have not been investigated, defects associated with defaults in Fe-S cluster assembly and trafficking in Δ*bol1-3*, Δ*iba57*, and Δ*isca1/2* yeast mutants were functionally complemented with Arabidopsis BOLA, IBA57, and ISCA proteins (Uzarska et al., 2018), suggesting that the corresponding plant proteins contribute to the assembly of an holo-complex I (absent in *Saccharomyces cerevisiae*). Concerning NFU1, contrasting results have been obtained since a complex I activity decrease to ∼30% was noticed in some patients (Cameron et al., 2011), but not reported in another study (Navarro-Sastre et al., 2011). In both cases, lipoic acid-dependent enzymes were affected. Accordingly, protein lipoylation was defective in Arabidopsis *nfu4 nfu5* lines unlike complex I activity (Przybyla-Toscano et al., 2022), likely excluding a role of NFUs for complex I assembly.

So far, INDH protein, also referred to as Ind1 or NUBPL in human, is the sole Fe-S cluster maturation factor specifically associated with complex I assembly. The IND1/INDH/NUBPL proteins belong to a subfamily of P-loop NTPases. *In vitro* reconstitution assays using *Yarrowia lipolytica* Ind1 and human IND1/NUBPL showed that they bind a [4Fe-4S] cluster using a conserved CxxC motif at the C-terminus (Bych et al., 2008; Sheftel et al., 2009). Genetic studies, either performed in HeLa cells by RNAi, with a *Y. lipolytica* deletion mutant or with Arabidopsis T-DNA insertion lines, evidenced a strong decrease in complex I protein and activity levels, and an accumulation of subcomplexes representing part or the totality of the membrane arm (Bych et al., 2008; Sheftel et al., 2009; Wydro et al., 2013), corroborating results in human patients presenting *IND1* mutations (Friederich et al., 2020). Importantly, the functional complementation of the *Y. lipolytica* deletion mutant by *Y. lipolytica* Ind1 variants recapitulating mutations found in human patients pointed to the accumulation of the Q module intermediate (Maclean et al., 2018). Overall, this indicates that INDH may be responsible for the delivery of [4Fe-4S] clusters to at least one subunit of the matrix arm. Analysis of the Arabidopsis *indh* mutant phenotypes also showed a translational defect affecting the matrix arm subunit Nad9, which suggests a role for INDH in mitochondrial translation as well (Wydro et al., 2013). Therefore, additional work is needed to clarify the exact role of INDH during complex I assembly.

## Conclusion

There are about 40 Fe-S proteins in Arabidopsis mitochondria present either in respiratory complexes or in the matrix (Przybyla-Toscano et al., 2021a). Only two types of proteins (ISU and GRXS15) should be involved in the maturation of [2Fe-2S] clusters and four types of proteins or protein couples (NFU, GRXS15/BOLA, ISCA/IBA57, INDH) in the maturation of [4Fe-4S] clusters. This indicates that these proteins likely contribute to the maturation of multiple acceptor proteins, but how specificity is achieved remains largely uncharacterized. For proteins integrated into larger complexes, it seems that Fe-S cluster insertion occurs in apo-proteins prior to their incorporation in complexes. Recent studies highlighted that complexes I–III share a common molecular mechanism at least for [2Fe-2S] cluster insertion. The current view is that the HSCB cochaperone recruits LYRM-bound target proteins for [2Fe-2S] cluster insertion by ISU proteins as exemplified by the requirement of LYRM7 for the Rieske subunit of complex III or of SDHAF1/SDHAF3 for complex II (Maio et al., 2017). The presence of additional LYR motif(s) in target proteins themselves likely participate in protein recognition as shown for complex II SDHB subunit (Maio et al., 2014). The maturation of [4Fe-4S] containing proteins is less clear but it should also require LYRM proteins and additional factors such as the complex I specific IND1/INDH maturation factors. The first example in plants is CIAF1 that may recruit TYKY for [4Fe-4S] cluster insertion by a yet unknown ISC maturation factor (Ivanova et al., 2019). In conclusion, the complete set of assembly and maturation factors required as well as the molecular mechanisms supporting the insertion of the different types of Fe-S clusters present in the peripheral arm remain largely elusive. In particular, biochemical and structural data are urgently needed to obtain direct evidence of (i) whether additional LYR-M proteins, either uncharacterized or yet unidentified, are necessary, (ii) which maturation factors are required for the insertion of the [4Fe-4S] clusters, and more generally (ii) how interactions between LYRM, maturation factors and acceptor proteins occur.

## Author Contributions

All authors have read and approved the manuscript.

## Conflict of Interest

The authors declare that the research was conducted in the absence of any commercial or financial relationships that could be construed as a potential conflict of interest.

## Publisher’s Note

All claims expressed in this article are solely those of the authors and do not necessarily represent those of their affiliated organizations, or those of the publisher, the editors and the reviewers. Any product that may be evaluated in this article, or claim that may be made by its manufacturer, is not guaranteed or endorsed by the publisher.
